# Supplementary material for: Constitutive Occurrence of E:N-cadherin Heterodimers in Adherens Junctions of Hepatocytes and Derived Tumors
Source: Cells. 2022 Aug 12;11(16):2507. doi: 10.3390/cells11162507 (PMC9406782; doi:10.3390/cells11162507)
Supplement: Supplementary file 1 [file cells-11-02507-s001.zip › cells-1801518-supplementary.pdf]

Supplementary Table S1: List of antibodies

Antibody	Supplier	Product Nr.	Type
Anti-AFP	Dako	IR500	rabbit polyclonal
Anti-alpha-Catenin	Invitrogen	13-9700	mouse monoclonal
Anti-beta-Catenin	BD Biosciences	610154	mouse monoclonal
Anti-CD34	Dako	GA632	mouse monoclonal
Anti-CK19	Dako	IR632	mouse monoclonal
Anti-E-cadherin	Epitomics	EP700Y	rabbit monoclonal
Anti-E-cadherin	BD Biosciences	610182	mouse monoclonal
Anti-Glypican 3 (GPC3)	Roche	790-4564	mouse monoclonal
Anti-HSP70	Santa Cruz	Sc-24	mouse monoclonal
Anti-Ki67	Dako	IR626	mouse monoclonal
Anti-N-cadherin	Calbiochem	42031	rabbit polyclonal
Anti-N-cadherin	BD Biosciences	13A9	mouse monoclonal
Anti-p120	BD Biosciences	610134	mouse monoclonal
Anti-Plakoglobin	Progen	61005	mouse monoclonal
Anti-ZEB1	Novusbio	NBP1-05987	rabbit polyclonal
Anti-ZO-1	Invitrogen	33-9100	mouse monoclonal
